# Relationship between beta-globin gene carrier state and insulin resistance

**DOI:** 10.1186/2251-6581-11-22

**Published:** 2012-11-19

**Authors:** Adele Bahar, Zahra Kashi, Mehrnoush Sohrab, Mehrnoush Kosaryan, Ghasem Janbabai

**Affiliations:** 1 Diabetes Research Center, Mazandaran University of Medical Sciences, Sari, Iran; 2 Deparment of internal medicine, Imam Khomeini hospital, Razi street, Sari, Iran; 3 Thalassemia Research Center, Mazandaran University of Medical Sciences, Sari, Iran; 4 Cancer center, Mazandaran University of Medical Sciences, Sari, Iran

**Keywords:** Thalassemia minor, Insulin resistance, Impaired glucose tolerance, Diabetes mellitus, HOMA

## Abstract

**Objective:**

To assess the relationship between being beta globin gene carrier and developing insulin resistance.

**Methods:**

This study was conducted on 164 subjects including 82 healthy ones and 82 patients with beta thalassemia minor (microcytosis (MCV <80 fl) and hypochromia (MCH <25 pg) and HbA2 ≥ 3.5% using HPLC). Fasting blood glucose (FBS) values of 100–125 mg/dl were considered as impaired fasting glucose, and above 125 mg/dl as diabetes mellitus. Two hours After 75 gram glucose load(GTT), blood sugar level of 140–199 mg/dl was considered as impaired glucose tolerance and above 199 mg/dl as diabetes mellitus. Insulin resistance was diagnosed based on homeostasis model assessment method (HOMA).

**Results:**

According to FBS and BS2hPG values, the percentages of diabetes mellitus, pre diabetes, and normal glucose tolerance in case group was 8.5%, 9.8% and 81.7%, respectively. There was no case of diabetes mellitus in control group and 6.1% of this group were pre diabetic and 93.9% of them had normal glucose tolerance test (P = 0.02). Relative risk for diabetes mellitus and insulin resistance in the cases with minor thalassemia was 2 (95% CI: 1.8-2.5) and 2.02 (95% CI: 1.7-2.4), respectively.

**Conclusion:**

The risk of developing diabetes and insulin resistance in patients with thalassemia minor is two times greater than the general population. Considering the high serum levels of CRP in these cases, the inflammation noted in liver cells could be considered as the underlying cause of insulin resistance, impaired glucose tolerance and diabetes in these patients.

## Introduction

Insulin resistance is a common problem in patients with different types of hemoglobinopathies including beta thalassemia major. Excessive iron deposition in the pancreas and liver of patients suffering from thalassemia major as well as insulin resistance has been described [1]. It is believed that the increase in iron overload may damage the pancreatic β cells [2]. Increased iron turnover, similarly, may damage liver, contributing to oxidative stress and insulin resistance [3]. The reported prevalence of impaired glucose tolerance and diabetes mellitus in thalassemia major is up to 24% and 26% respectively and it is believed that insulin resistance can be occurred in these patients before the onset of glucose intolerance or diabetes mellitus [4]. Insulin resistance in beta thalassemia minor (carrier state) has been demonstrated as well [4].

Considering high number of complications associated with insulin resistance and impaired glucose metabolism, the present study was designed to evaluate the prevalence of these metabolic disorders in patients with beta thalassemia minor.

## Materials and methods

After being approved by the Ethical Board of Mazandaran University of Medical Sciences, Sari, Iran this study was conducted on 82 individuals with beta thalassemia minor and 82 healthy subjects as control group[according to P1=6% (prevalence of IGT in normal population), P2=14% (prevalence of IGT in beta thalassemia minor), d_2_=10% , Z_1_-β ,Z_1_- α/_2_=1.96]. The diagnosis of beta thalassemia minor was based on the detection of hemoglobin (Hgb) A2 > 3.5% by column chromatography (Spain bio system Co.) and hemoglobin electrophoresis using HPLC (High-performance liquid chromatography) and cellulose acetate gel.

Subjects in the two groups were matched in terms of body mass index (BMI) and family history of diabetes mellitus. Age range was from 20 to 60 years of age. Blood pressure values were normal (having diastolic blood pressure < 80 mmHg and systolic blood pressure < 120 mmHg) [5].

Exclusion criteria consisted of history of kidney, liver and heart disease, history of diabetes, receiving medications which affect carbohydrate metabolism (such as corticostroids,cyclosporin,closapin) and waist circumference (WC) more than 102 cm in men and more than 88 cm in women [6].

Anthropometric measurements including weight and height were obtained with light clothing and without shoes by trained technicians following international guidelines. The height (to the nearest 0.1 cm) and weight (to the nearest 0.1 kg) were measured using a stediometer and a digital scale (Micro life ws80, Switzerland), respectively. The BMI was calculated as the body weight divided by the height squared (Kg/m^2^). WC was measured using a non-elastic flexible anthropometric tape (to the nearest 0.1 cm) in the standing position. The tape was applied horizontally midway between the lowest rib margin and the iliac crest.

A fasting blood sample (FBS) was taken from all the participants. Serum FBS was analyzed by enzymatic calorimetric method using glucose kit of Parsazmoon Co, Iran. AST (Aspartate aminotransferase) and ALT (Alanine aminotransferase) were measured by calorimetric method using Biosystem kit, Spain and Hitachi917, Germany. High sensitive C-reactive protein (hs-CRP) was measured by imminotorbidometry assay by Pars Azmoon kit, England, using Hitachi917 machine Germany. ELISA test with Hitachi auto analizer using Monobind kit, USA was used to assess serum insulin levels. A standard oral glucose tolerance test (OGTT) was performed in all subjects.

Individuals with abnormal FBS (FBS ≥100) and/or abnormal OGTT (blood sugar 2 hours post-prandial (BS 2hpp) ≥140) were re-tested. FBS values below 100 mg/dl were considered as normal, 100–125 mg/dl as impaired fasting glucose, and above 125 as diabetes. BS 2hpp below 140 mg/dl was considered as normal, 140–199 mg/dl as impaired glucose tolerance and above 199 as diabetes [7].

Insulin resistance was calculated using HOMA-IR (homeostasis model assessment method) [8].

(1) HOMAIR=Fasting plasma insulin mIU/L×Fasting Plasma Glucose mmol/l22.5

Considering HOMA-IR values, the studied population was divided into three groups:

1. (Insulin Sensitive) HOMA-IR < 2.24

2. (Intermediate) 2.24 ≤ HOMA-IR ≤ 3.59

3. (Insulin Resistance) HOMA-IR > 3.59

The student t-test and chi-squared test were used to compare demographic data between the two groups. Relative Risk was measured to test the risk of diabetes and insulin resistance in two groups. P value less than 0.05 was considered statistically significant.

## Results

In patient group, 53 cases (64.6%) were female and 29 (35.4%) were male. In control group 38 subjects (46.3%) were male and 44 (53.7%) were female (P = 0.1). Other demographic data are outlined in Table 1: According to FBS and BS2hPG values, the percentages of cases with diabetes mellitus, impaired fasting glucose, and normal glucose tolerance were 8.5%, 9.8% and 81.7%, respectively. There was no case of diabetes mellitus in control group and 6.1% of subjects had impaired fasting glucose and 93.9% of them were normal glucose tolerance test. (P = 0.02).

**Table 1:** Demographic findings of the subjects with beta thalassemia minor regarding demographic data, Sari, Iran, 2010

		**Controls**	**Cases**	**P value**
		**(N = 82)**	**(N= 82)**	
		**Mean ± SD**	**Mean ± SD**	
Age (year)		39.5±8.7	39.7±8.8	0.8
Weight(kg)		67.4±8.5	66.4±8.4	0.4
Height(meter)		170.1±7.7	169.3±7.5	0.5
BMI(kg/m^2^)		22.9±1.4	22.7±1.4	0.4
Systolic blood pressure(mmHg)		109±4.9	110±4.9	0.6
Diastolic blood pressure(mmHg)		67±4.9	68±5.4	0.8
Waist(cm)	Male	83±2	83±2.2	0.8
	Female	77±1.3	76±1.8	0.8

According to FBS and BS2hPG values, the percentages of cases with diabetes mellitus, impaired fasting glucose, and impaired glucose tolerance were 7.3%, 11% and 81.7%, respectively. There was no case of diabetes mellitus in control group and 7.3% of subjects had impaired fasting glucose or impaired glucose tolerance. (P = 0.02).

AST and hs-CRP values were also similarly higher among the cases (Table 2). There was, however, no significant difference between mean ALT, fasting insulin and HOMA-IR levels between the two groups .Fasting insulin level was 6.5± 5.3 and 5.9±2.0 in case and control group respectively.

**Table 2:** Laboratory values of liver enzymes, CRP, fasting insulin, HOMA-IR in each group

	**Controls**	**Cases**	**P value**
	**(N = 82)**	**(N= 82)**	
	**Mean ± SD**	**Mean ± SD**	
AST (IU/L)	20.9 ± 7.3	23.6 ± 8.1	0.03
ALT (IU/L)	15.5 ± 7.5	18.3 ± 12.8	0.09
CRP (mg/L)	2.97 ± 1	3.9 ± 3.2	0.01
Fasting serum insulin (μIU/ml)	5.9 ± 2	6.5 ± 5.3	0.4
HOMA-IR	1.1 ± 0.4	1.2 ± 0.4	0.7

Relative risk of developing diabetes mellitus (based on FBS and BS2hPG values) and insulin resistance(based on HOMA-IR) in patients with beta thalassemia minor were 2 (CI 95%: 1.8-2.5) and 2.025 (CI 95%: 1.7-2.4) respectively.

## Discussion

In line with previous studies, our findings revealed that the relative risk of developing diabetes mellitus and insulin resistance in patients with beta thalassemia minor is two times higher than the general population. Tang et al. reported that thalassemia minor patients with normal glucose tolerance have higher fasting insulin levels and insulin resistance (HOMA-IR) than the healthy controls matched for age, gender and BMI [4]. In another study Alhezmy et al. reported that 6 percent of thalassemia major patients and only 2 percent of the kids with thalassemia minor or no hemoglobinopathy were diagnosed with diabetes mellitus. The prevalence of impaired glucose tolerance was, similarly, higher in patients with thalassemia major compared to those suffering from thalassemia minor and healthy subjects. In this study, subjects were not matched in terms of BMI and family history of diabetes; both of which play an important role in the development of diabetes and insulin resistance [9].

Corroborating previous studies, our research reported significantly higher CRP levels in thalassemia minor patients [4]. This finding can explain the higher prevalence of insulin resistance in these patients [10]. Mean AST and ALT levels were also higher in this group of patients [11]. The correlation between ALT levels and insulin resistance has been reported in other studies [12-15]. Berget et al. while studying a group of adolescents, pointed out that any increase in ALT levels, even when it is within the normal range, lowers the sensitivity of the cells to insulin, contributing to impaired glucose tolerance and increased levels of free fatty acids and triglycerides [11]. In another study in Native American adults (Pima Indians), ALT was reported as a predictor of type II diabetes [15]. Considering the higher serum levels of liver enzymes and hs-CRP in thalassemia minor patients, the inflammation of liver cells could be accounted for the underlying cause of insulin resistance and impaired glucose tolerance in these patients [4]. In other words, liver inflammation and increased oxidative stress secondary to microcytic erythrocytes hemolysis is the main reason contributing to higher prevalence of diabetes in thalassemia minor patients. According to this concept Noetzli et al. in their study showed that Pancreatic iron overload and diabetes mellitus (DM) are common in thalassemia major patients and iron deposition in pancreas is the strongest predictor of beta cell toxicity and total body iron burden, age, and body habitus also influence glucose regulation too [16].

The certain limitation of our study was that serum level of ferritin a marker of liver inflammation was not measured though we measured the CRP another marker of inflammation.

## Conclusion

The present study revealed that similar to thalassemia major sufferers, the prevalence of insulin resistance and diabetes is higher in patients with thalassemia minor than the general population. Considering the high prevalence of thalassemia minor in our country and the fact that impaired glucose metabolism is associated with multiple complications, it is recommended to screen thalassemia minor patients for glucose tolerance at a younger age. Further studies are also required to detect other mechanisms contributing to insulin resistance in these patients.

## Competing interests

We have no competing interests.

## Authors' contributions

AB: ES-FG, ZK: ES-FG, MS: ES-FG, MK: FG, GJ: FG. All authors read and approved the final manuscript.
